# Iliac Crest Avulsion Fracture in a Young Sprinter

**DOI:** 10.1155/2015/302503

**Published:** 2015-09-01

**Authors:** L. Casabianca, R. Rousseau, P. Loriaut, A. Massein, G. Mirouse, A. Gerometta, F. Khiami

**Affiliations:** ^1^Department of Orthopaedic and Sport Surgery, Pitié Salpêtrière Hospital, Pierre and Marie Curie University, 47 Boulevard de l'Hôpital, 75013 Paris, France; ^2^Department of Radiology, Pitié Salpêtrière Hospital, Pierre and Marie Curie University, 47 Boulevard de l'Hôpital, 75013 Paris, France

## Abstract

Avulsion fracture of the iliac crest is an uncommon pathology. It usually occurs in teenagers during sport activities, more common in boys. We report a case of 16-year-old male competitive sprinter, who had an avulsion of a part of the iliac crest and the anterior-superior iliac spine during a competition. The traumatism occurred during the period of acceleration phase out of the blocks which corresponds to the maximum traction phase on the tendons. Then a total loss of function of the lower limb appears forcing him to stop the run. X-ray and CT scan confirmed the rare diagnosis of avulsion of the quasitotality of the iliac crest apophysis, corresponding to Salter 2 fracture. We performed an open reduction and internal fixation with two screws, allowing a return to sport after 3 months and his personal best record in the 100 meters at the 6th postoperative month.

## 1. Introduction

Avulsion fractures of the pelvic apophyses are uncommon, with mean age of 14.4 years, represented by the anterosuperior iliac spine (ASIS) 49%, anteroinferior iliac spine (AIIS) 30%, ischial tuberosity 11%, and the iliac crest [1]. Avulsion fracture of the ASIS represented 1.4% of hip and pelvis injuries [2]. It is usually caused by sudden strain or unbalanced contraction on the sartorius or the tensor of the fascia lata [3]. They are most commonly seen affecting the growing apophyses of teenagers and are often missed on initial presentation.

The purpose of this case report is to present a rare pathology of iliac crest avulsion and clarify the mechanism of trauma in sprinters and explain how a surgical treatment can obtain an anatomical result with a return to sport in the best possible conditions in a high-level athlete patient.

## 2. Case Report

The patient is a 16-year-old male sprinter; he had no particular medical history. He participates in national and international competitions; the frequency of his training was 5 per week of 3 hours. During those 3 hours he dedicates 30 minutes for the warm-up, but he did not do stretching exercises.

The sprinter felt pain during the acceleration phase out of the blocks. There then a total loss of function of the lower limb appeared forcing him to stop running.

He was checked out by a doctor of the Athletics Federation a few days afterwards. A physical examination revealed an anterior pain located on the anterior spine and a total loss of function of the hip.

**Figure 1:**
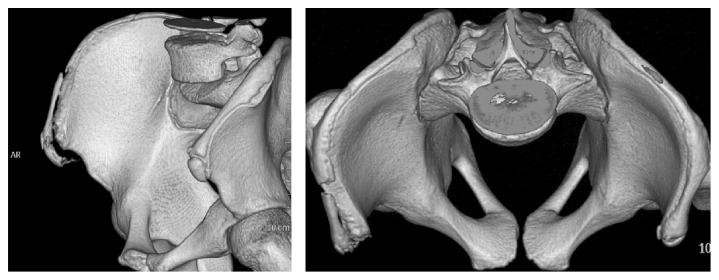
3D CT scan. avulsion of a part of the iliac crest apophysis and the ASIS, downward and more outward.

X-ray and CT scan confirmed the rare diagnosis of avulsion of a part of the iliac crest apophysis (Figure 1). We made a preoperative CT scan to identify the lesions and to calculate precisely the displacement of the fracture. The displacement of the tear was large: 9 mm downward and more outward.

This partial crest injury corresponds to the part of this apophysis which is still not close. The stage of bone maturation of his iliac crests corresponds to a Risser stage 3. The Tanner stage of the patient is between 3 and 4.

**Figure 2:**
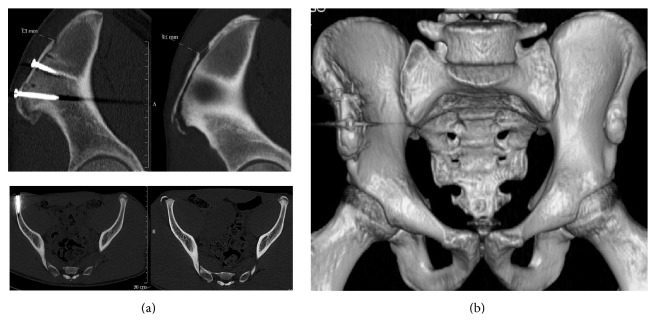
CT scan. (a) before (left) and after operation (right): reduction and internal fixation with two screws of the avulsion. (b) 3D CT scan. Good position of the fragment with two screws.

We performed an open reduction and internal fixation with two screws. The CT scan control confirmed the reduction and good position of the screw (Figure 2).

The postoperative instructions were no weight bearing for 6 weeks without immobilization and no active flexion of the hip. The patient then began muscular reinforcement and proprioception with a physiotherapist and equalled his personal best record in the 100 meters at the 6th postoperative month. No postoperative complication was noted.

## 3. Material and Methods

### 3.1. Generality

The average age of apophyseal avulsions in the pelvis is 14,4 years within a range of 11–17 years [1]. During this period, apophyses, where strong muscles are inserted, are the weakness zone of the musculoskeletal apparatus of young people. This weakness is reflected in the fragility of the enchondral ossification of the apophyses facing biomechanical constraints exerted by much stronger and more resistant muscles. The lesions are usually due to a sudden increase (sudden, violent, concentric, or eccentric) in tension during high sporting activities in subjects with an immature skeleton [4]. Among the apophyseal avulsions in the pelvis, avulsion of the ASIS is the most common, followed by avulsion of the ischial tuberosity and then by the anterior inferior iliac spine [4–8]. Avulsion of a part of iliac crest is a very rare pathology and few are described in the literature.

Sports activities responsible for the anterior superior iliac spine avulsion fractures were soccer, athletics, and gymnastics [4].

Two muscles start at the ASIS—the sartorius and the tensor fascia lata—and on the iliac crest—the fascia lata, the transverse abdominal muscle, and the internal oblique abdominal muscle [5]. This is why White described two types of injuries depending on the sport, topography, and lesion size for ASIS avulsions.

Type I sartorius avulsion fracture is due to sprinting in various sports; the fragment is smaller and displaced anteriorly [9].

Type II rarer, tensor fascia lata avulsion fractures are due to swinging a baseball bat. The two muscular sartorius and the tensor fascia lata are both injured during the initial phase of batting. The bony fragment is much larger and displaced laterally [9].

**Figure 3:**
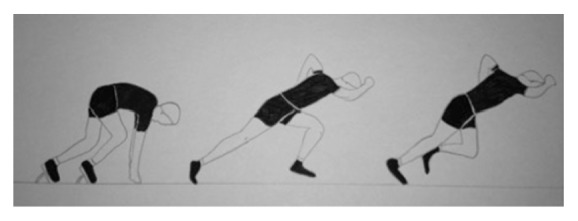
Propulsion at the exit of starting block. Hip extension and maximum knee extension, combined with a slight rotation of the trunk, corresponding to a traction on sartorius and fascia lata associated with a traction on abdominal muscle explaining the large lesion.

In our case it was a type 2 with an extension to the iliac crest that occurred during the acceleration phase out of the blocks in a sprinter, which does not completely concur with White's theory. The patient described pain during propulsion at the exit of starting block: this is the acceleration phase out of the blocks which corresponds to the maximum traction phase on the tendon during the run. The tear was made during hip extension and maximum knee extension, combined with a slight rotation of the trunk corresponding to the maximum traction on the sartorius and the fascia lata and combining abdominal muscles traction (Figure 3). This explains why the avulsion was larger and laterally displaced (Figure 1).

### 3.2. Clinical Diagnosis

Clinically, intense pain is the main symptom with little external evidence of trauma. Then a total loss of function of the lower limb appears. During the physical examination a tumefaction of the ASIS can be found. Palpation of this area triggers intense pain. Sometimes, the avulsed fragment is palpated under the skin. Rare initial presentation of ASIS fracture as meralgia paresthetica has been reported. The mechanism is not clear: this may be due to a hematoma entrapment of the inguinal ligament or perhaps a traction force on the nerve or an oedema [10, 11].

### 3.3. Radiological Diagnosis

The diagnosis of these lesions was done by plain radiographs with sometimes three quarters. They may be missed in conventional plain X-rays of the pelvis [12], so they must be completed in larges displacements by CT to specify the fragment size and the size of the dislocation. MRI examination is a more sensitive method for evaluating this injury when X-ray findings are inconclusive [12].

## 4. Discussion

There Are Many Opinions about Current Treatments for ASIS.

The conservative treatment generally consists of bed rest or being on a chair for a period of 3 weeks, with the affected hip at a 70° flexion. Symptomatic treatment of pain and anti-inflammatory treatment should be given. After three weeks, one could carefully begin physiotherapy and ambulation with crutches. Partial weight bearing using crutches is advised until six weeks after injury and from then on gradual full weight bearing.

The operative treatment consists of open reduction and internal fixation by screws using a standard anterior approach; weight bearing is allowed immediately after the drain is removed. Hospitalization time is a few days. After 6 weeks, full weight bearing is allowed.

The indication for the operative treatment that we found in the literature was fracture displacement >2 cm that is advocated when a short recovery time is desired [7, 13]. Moreover, meralgia paresthetica is another indication that operative treatment is needed [11].

The benefit of operative treatment is allowing a better and faster bone healing. At six weeks, Kautzner et al. found 76% of patients in the surgical group with signs of good healing and fragment integration compared to only 50% in the conservative group [3].

For some experts this treatment should remain an exception and should be reserved for patients for which early resumption of intense activity level is necessary or large displacement [3].

The complications of the conservative treatment are represented mainly by nonunion and exostoses [14]. This is why massage and active rehabilitation are proscribed for six weeks. Most patients recover completely without complications after conservative treatment [9, 15].

But the complications of surgical treatment are rare deep infection, prolonged wound healing, keloid scars, and transient hyperesthesia of the lateral femoral cutaneous nerve [3].

Concerning heterotopic ossification, there is no difference between surgical and nonoperative groups. In the same study at the one-year follow-up, all patients had the same level of bone healing [3].

## 5. Conclusion

Our advice to avoid this kind of lesions is to not neglect the warm-up, do preparatory work before the main effort, and then relax and stretch muscles. Avulsion fracture of the ASIS is a rare injury and iliac crest avulsion is even rarer. Both treatment options give good long-term results. In our opinion, surgery is needed in large displacements with extension to the iliac crest and if the patient is an athlete since it allows a fast recovery. In this case, the significant tearing of almost all the iliac crest is an additional argument for surgery.
